# Quantifying Accelerations and Decelerations in Elite Women Soccer Players during Regular In-Season Training as an Index of Training Load

**DOI:** 10.3390/sports9080109

**Published:** 2021-07-31

**Authors:** Tom Douchet, Allex Humbertclaude, Carole Cometti, Christos Paizis, Nicolas Babault

**Affiliations:** 1 Center for Performance Expertise, CAPS, U1093 INSERM, Sport Science Faculty, University of Bourgogne-Franche-Comté, 3 Allée des Stades Universitaires, BP 27877, CEDEX, 21078 Dijon, France; tom.douchet@gmail.com (T.D.); a-humbertclaude@laposte.net (A.H.); carole.cometti@u-bourgogne.fr (C.C.); christos.paizis@u-bourgogne.fr (C.P.); 2 Dijon Football Côte d’Or (DFCO), 17 rue du Stade, 21000 Dijon, France

**Keywords:** workload, monitoring, sprint, heart rate, rate of perceived exertion, GPS

## Abstract

Accelerations (ACC) and decelerations (DEC) are important and frequent actions in soccer. We aimed to investigate whether ACC and DEC were good indicators of the variation of training loads in elite women soccer players. Changes in the training load were monitored during two different selected weeks (considered a “low week” and a “heavy week”) during the in-season. Twelve elite soccer women playing in the French first division wore a 10-Hz Global Positioning System unit recording total distance, distance within speed ranges, sprint number, ACC, DEC, and a heart rate monitor during six soccer training sessions and rated their perceived exertion (RPE). They answered the Hooper questionnaire (sleep, stress, fatigue, DOMS) to get an insight of their subjective fitness level at the start (Hooper S) and at the end of each week (Hooper E). A countermovement jump (CMJ) was also performed once a week. During the heavy week, the training load was significantly greater than the low week when considering number of ACC >2 m·s^−2^ (28.2 ± 11.9 vs. 56.1 ± 10.1, *p* < 0.001) and number of DEC < −2 m·s^−2^ (31.5 ± 13.4 vs. 60.9 ± 14.4, *p* < 0.001). The mean heart rate percentage (HR%) (*p* < 0.05), RPE (*p* < 0.001), and Hooper E (*p* < 0.001) were significantly greater during the heavy week. ACC and DEC showed significant correlations with most outcomes: HR%, total distance, distance per min, sprint number, Hooper index of Hooper E, DOMS E, Fatigue E, RPE, and session RPE. We concluded that, for elite women soccer players, quantifying ACC and DEC alongside other indicators seemed to be essential for a more complete training load monitoring. Indeed, it could lead to a better understanding of the reasons why athletes get fatigued and give insight into neuromuscular, rather than only energetic, fatigue.

## 1. Introduction

The use of data has revolutionized sport and, in particular, training periodization. Nowadays, the choices of the technical staff should be supported by data-driven evidence. Moreover, this approach became more popular due to the increased reliability/validity [1] and miniaturization of wearable devices that enable real-time monitoring. Real-time monitoring quickly became popular in elite sports and, in particular, in soccer, with widespread use across the majority of top-level European teams [2]. Quantifying training loads achieved by players in order to get insight into their responses to training and preventing injuries is crucial [3]. For instance, this feedback allows the technical and physical staff to keep players in an optimal condition and, in turn, reduce injury risk [4].

To quantify training load during soccer training sessions or matches, the majority of studies have focused on assessing the global demand using, for instance, the total distance covered within predefined speed ranges [5,6,7]. The ability to assess these demands has widely helped our understanding of soccer matches and training sessions. However, it excludes specific fundamental actions, such as direction changes, accelerations (ACC), and decelerations (DEC). These actions, which depict the rate of change of velocity with respect to time [8] are part of the high-intensity external load (distance covered) performed by elite players. Therefore, excluding ACC and DEC in players’ monitoring could potentially underestimate the stress of this sport. When including ACC and DEC during video analysis of soccer matches alongside distance covered within speed ranges, a 6–8% increase of the training load has been highlighted [9]. Indeed, “high-intensities” expressed as high-power output actions are two to three times larger when considering ACC and DEC in match analysis than when only considering running activities [9]. It has been shown in professional football players that the longer the time spent accelerating and decelerating, the greater the rating of perceived exertion (RPE) and the concentration in blood lactate [10]. These data revealed that ACC and DEC are very demanding actions even at submaximal speeds. Moreover, the quantification of these actions may provide an insight into the neuromuscular fatigue generated by training. Authors have previously shown that an increase in the number of ACC and DEC led to greater increases in plasma creatine kinase [11]. In this regard, creatine kinase has been associated with decreased performance [12] and increased injury risks in soccer [13].

Given the importance of ACC and DEC, and that very few studies have considered elite women players, this study aimed to investigate whether quantifying ACC and DEC could help to monitor training load in elite women soccer players. Increasing our understanding of training load monitoring could help to avoid overreaching/overtraining and increase player health status by decreasing the injury risk. For that aim, two different weeks with varying training load and intensity (as planned by the technical staff) were considered for analyses. We hypothesized that these actions were sensitive enough to discriminate training load and were significantly associated with the majority of the indicators usually monitored. These associations would demonstrate that ACC and DEC play an important role in players’ training load. Moreover, we hypothesized that a week with more ACC and DEC would induce a drop of the readiness level.

## 2. Materials and Methods

### 2.1. Subjects

**Figure 1:**
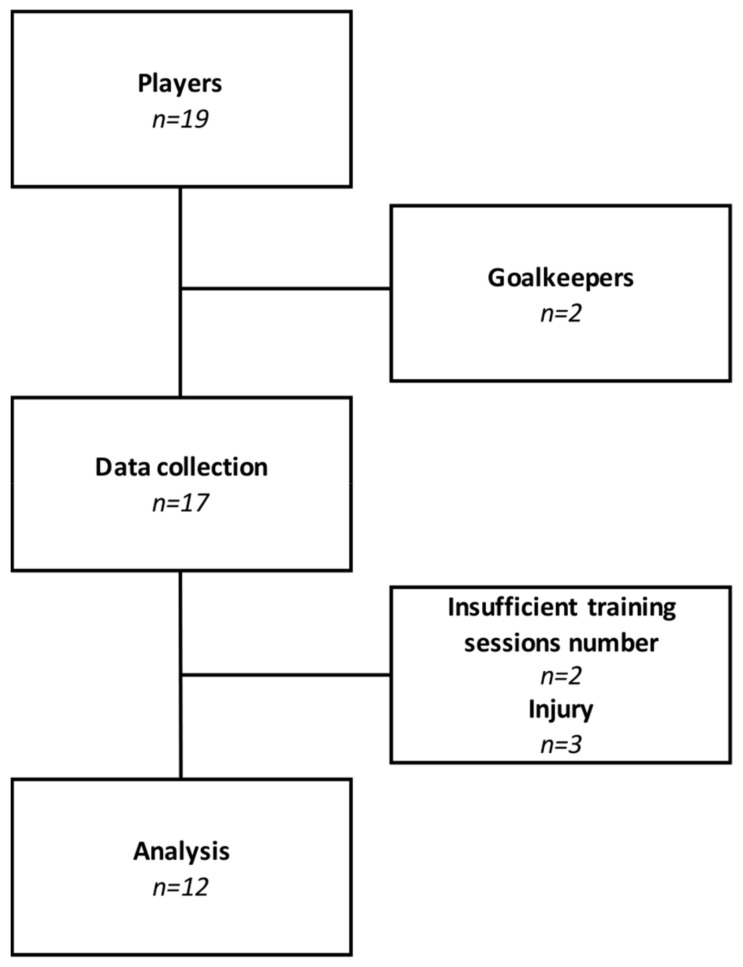
Study flowchart. The team consisted of 19 players. Goalkeepers and players with insufficient training numbers were removed from analyses. Injuries represent two sprained ankles and one strained hamstring.

Nineteen elite national-level women soccer players (professional) competing in a French first division team (age = 24.2 ± 2.3 years; height = 169.7 ± 5.2 cm; body mass = 62.3 ± 5.1 kg) were included. Goalkeepers were excluded due to the different nature of their activity. Players with insufficient training sessions were excluded (less than 60% of completed sessions) but still participated in other sessions. Twelve players were therefore considered for analyses (Figure 1). All players were notified of the research protocol, benefits, and risks before providing a signed written informed consent in accordance with the Declaration of Helsinki. Approval of the study was obtained from the local ethics committee.

Training load was quantified during two different weeks within the first part of the competitive season. Weeks were a priori chosen according to the technical and physical staff in order to conduct measurements during low (low week) and high training-load (heavy week) weeks. Accordingly, week 13 (low week) and week 16 (heavy week) were monitored during the 2018–2019 season. These weeks were part of the same 4-week voluminous conditioning mesocycle. The low week aimed to include some rest as a result of a preceding intensive conditioning mesocycle. The heavy week corresponded to high training load (either technical and physical) coincident with the end of the voluminous conditioning mesocycle.

Weekly training organization is presented in Table 1. Training included four main training sessions with three field-based sessions (Tuesday, Wednesday, and Friday evening) and one gym-based strength training session (Tuesday morning). The three field-based training sessions consisted of technical, tactical, and/or physical training. The low week consisted of two technical and tactical sessions (during training 1 and 3) and one technical and physical session (during training 2). The heavy week consisted of a single technical and tactical session during training 1 and two technical and physical training sessions (during training 2 and 3). The two gym-based strength training sessions were identical and consisted of a global warm-up containing sub-maximal contractions of the different muscle groups implied during training, followed by squat and deadlift for 4 sets of 6 maximal repetitions. All technical and physical sessions were conducted on an artificial grass field, and the main work consisted of 4 vs. 4 plus goalkeepers small-sided games on a 30 × 40 m field representing 150 m^2^ per player. In addition, a strength training session, power development-oriented, was conducted during the two weeks considered. These sessions were similar. The warm-up period prior to each training session was included for analysis. No individual rehabilitation session was included for analysis. When players were rested by the coaching staff or injured, training sessions were not considered, and such players were therefore excluded from analysis (Figure 1).

### 2.2. Procedure

During the two weeks, a total of 72 (36 for each week) individual training observations were undertaken on the 17 outfield players from the 102 possible. The study included the players who completed at least 60% of all the training monitored (12 players). It represented five defenders (*n* = 30), three midfielders (*n* = 18), and four attackers (*n* = 24). To be able to compare the two monitored weeks, players had to perform the same number of training sessions in both weeks. Due to the inability to wear GPS devices in professional matches due to regulations, matches were not included in this study.

The players’ training load during each training sessions was monitored using a Polar Team Pro sensor [14] (Polar Electro, Kempele, Finland), which simultaneously recorded spatial position (GPS) and heart rate (HR) [15]. As specified by the manufacturer, the sensor combines signals from a 10 Hz GPS with a 200 Hz sampling frequency microelectromechanical inertial measurement unit consisting of a tri-axial accelerometer, gyroscope, and magnetometer. The sensor was attached to the skin approximately above the xiphoid process through a typical chest band for HR monitor. To avoid any data loss due to possible latencies (e.g., satellite signals reception), all devices were activated 15 min before the beginning of the training session. The electrode area of the straps was moistened with water to achieve an optimal connection. The sensors were distributed to players 10 min before the start of the session and started recording as soon as a HR signal was detected and internally registered data. Once the coaching staff reunited the players at the end of the training session, the GPS was turned off. GPS microsensors were then synchronized to the Polar Team Pro station. We used the time of the day to manually set the start and end of the training session on the Polar software. The sessions were taken into account, including the warm-up and up to the final whistle of the coach. If a player realized extra-work as part of the coaching staff plan, it was included into analysis.

HR (expressed in beats per minute, bpm) was monitored as an indicator of the internal load. For each individual, we defined: 1. maximal HR (the maximal value recorded during the two weeks) and 2. mean HR percentage (mean HR%) during each training (expressed as a percentage of the maximal HR recorded during the two weeks). As maximal aerobic speed (obtained through an incremental intermittent test earlier in the season [16]) work was implemented during these weeks, we can assume that maximal heart rate was reached.

The external load was quantified using the total distance expressed in meters (m). The total distance covered corresponded to every meter covered from walking to sprinting. This value was expressed relative to the training duration in m.min^−1^ (m.min^−1^) as an index of the training session intensity. In addition, the distance was calculated with different speed zones (SZ). Five different SZ were used as predefined within the polar system: SZ1 (walking < 8.00 km·h^−1^), SZ2 (jogging between 8.00 and 12.00 km·h^−1^), SZ3 (low speed running between 12.00 and 15.00 km·h^−1^), SZ4 (moderate speed running between 15.00 and 18.00 km·h^−1^) [17], and SZ5 (high speed running more than 18.00 km/h). We also quantified the number of sprints (sprints with acceleration >2.8 m·s^−2^ for more than 1.5 s). We also quantified the total number of ACC (>2 m·s^−2^) and the number of DEC (<−2 m·s^−2^) using the same thresholds as previous female-soccer research [18]. All the GPS-derived indicators were considered as a sum of each training, in order to have a weekly total. Statistics were then made considering the weekly total.

RPE (CR-10 scale) [19] was collected between 15 to 30 min [20] after each training session (field-based or strength session) on individuals’ smartphones [21] to ensure that the perceived effort was referred to the whole session rather than the most recent exercise intensity. They had to answer to the following question: “How hard was the training session?” The aim of answering on their smartphone was to prevent players from giving the same value as their partners. We also quantified session RPE (sRPE), which is a simple way to quantify internal and external load [22]. It was calculated as the RPE multiplied by the training duration in minutes from the beginning of the warm-up and until the coach’s final whistle (arbitrary units).

At the beginning of each week, on Tuesday morning, just before the gym-based strength session, a countermovement jump (CMJ) was performed to assess any potential neuromuscular fatigue produced by the preceding week and match [23]. Players had to jump as high as possible beginning on a standing position, then flexing the knees until 90° and extended the knees to jump in a continuous movement. They were asked to keep their arms on their hips from the standing position until landing. Players were supervised by a conditioning coach to ensure proper execution, and knee angulation during flexion was monitored by video. If execution was poor, the player had to repeat the procedure. Prior to the jump, a standardized warm-up was realized, including 10 standing calf raises, 10 full squats, 6 concentric (the player laid on her stomach and tried to bend her leg at the knee against resistance) and 6 eccentric (the player laid on her stomach and tried to stop the extension of the leg at the knee against resistance) repetitions per leg, on the hamstrings. Jump flight time was measured using a photocell jump system (Optojump Next, Microgate, Bolzano, Italy) sampling at 1000 Hz, with jump height (cm) subsequently calculated by proprietary software (Optojump Next, Version 1.3.20.0, Microgate, Bolzano, Italy) [24]. Two jumps were realized with 60 s rest between, and the best was kept as the week value.

Players had to complete the Hooper Questionnaire, as a subjective value at the start of the week, prior to the first session (Hooper S) and at the end of the week on Saturday morning (Hooper E). This questionnaire detects signs of pre-fatigue and stress [25]. Athletes provided information about their subjective assessment of sleep quality the previous night, as well as ratings of fatigue, stress, and muscle soreness (DOMS). Items were noted as follows Sleep S, Stress S, Fatigue S, DOMS S, and Sleep E, Stress E, Fatigue E, and DOMS E, for the first and second questionnaire of the week, respectively. Each response was rated on a seven-point Likert scale, with responses ranging from “very, very good = 1” to “very, very bad = 7” for sleep, and from “very, very low = 1” to “very, very high = 7” for fatigue, stress, and DOMS. The Hooper Index (HI) was the summation of the four ratings.

### 2.3. Statistical Analyses

Statistical analyses were conducted using STATISTICA (Version 12.1, StatSoft, Tulsa, OK, USA). Results are presented as players’ week mean values (±standard deviation, SD), representing the three field-based sessions. As normality was verified using the Shapiro–Wilk test, parametric statistics were applied for all indicators. Student’s t-tests were used to analyze differences between the two weeks and Cohen’s d effect sizes were calculated to determine the association between the different outcomes with their interpretation based on the following criteria: <0.2 = trivial, 0.2 to 0.59 = small effect, 0.6 to 1.19 = moderate effect, 1.2 to 2.0 = large effect, and >2.0 = very large [26]. The statistical significance was set at *p* < 0.05. In addition, Pearson’s product moment correlation coefficients were associated with 95% confidence intervals (95% CI). Correlations were only made for ACC and DEC with all indicators in this study, except for HI Hooper S and its items and CMJ. Considering that these indicators were used in order to observe if players were in a comparable readiness level at the start of the week, correlations with ACC and DEC were not necessary.

## 3. Results

The two weeks under investigation started with players in a similar physical readiness level as attested by the similar CMJ and HI Hooper S at the beginning of the two weeks (Table 2 and Table 3).

The results indicated that the total number of ACC and DEC were greater during the heavy week than during the low week (*p* < 0.001) (Table 2). Similarly, the mean HR%, total distance, m·min^−1^, RPE, sRPE (Table 2), and the HI Hooper E (Table 3) were significantly greater during the heavy week compared to the low week (*p* < 0.005). While no difference was obtained for HI between the start and end of the week during the low week, it significantly increased during the heavy week (*p* < 0.001). For Hooper items, there were significant differences (*p* < 0.001) between the start and the end of the heavy week for Sleep, Fatigue, and DOMS. No difference existed for Stress. The comparison between the two weeks revealed some differences (*p* < 0.05) for Sleep E, Fatigue E, and DOMS E. No difference was observed for Stress E (Table 3). Also, sprint number showed no significant difference between the two weeks, while some significant differences can be highlighted between the two weeks for speed zones. Distances at SZ1, SZ2, SZ3, and SZ4 were greater (*p* < 0.05) during the heavy week compared to the low week. Distance at SZ5 showed no significant difference between the two weeks (Table 2).

**Figure 2:**
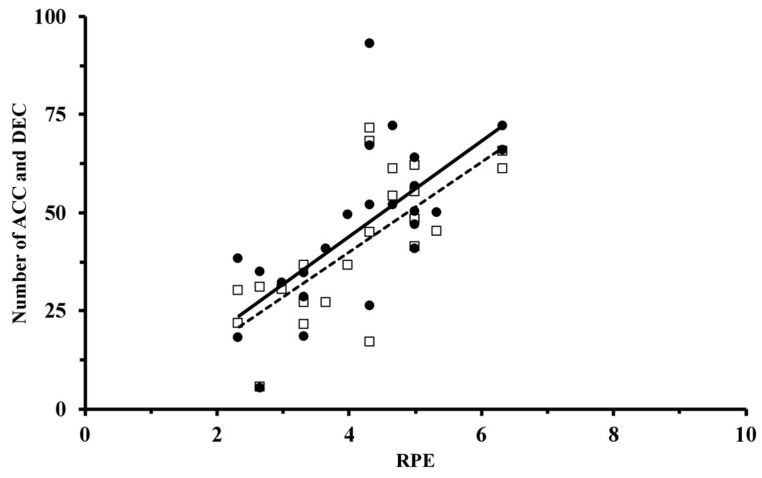
Correlation between the number of accelerations (ACC) >2 m·s^−2^ (white squares), the number of decelerations (DEC) < −2 m·s^−2^ (black circles), and the rate of perceived exertion (RPE). Each dot represents a player in one week, as values are presented as mean values of the week.

ACC showed significant correlations with almost all indicators monitored in this study (Table 4). Concerning the Hooper questionnaire (*p* < 0.05), ACC was correlated with HI Hooper E, Fatigue E, and DOMS E, while no significant correlations were established with Sleep E and Stress E. For external load indicators, ACC showed significant correlations (*p* < 0.05) with total distance; m·min^−1^; sprint number; and distance in SZ1, SZ2, SZ3, SZ4, SZ5, and DEC. For internal load, ACC demonstrated correlations (*p* < 0.05) with mean HR% and RPE (Figure 2). The combination of internal and external loads, represented by sRPE, was significantly correlated with ACC (*p* < 0.001).

For DEC, comparable results can be highlighted (Table 4). DEC was correlated with a major part of the Hooper items (*p* < 0.05) with HI Hooper E, Fatigue E, and DOMS E, while no significant correlations were established with Sleep E and Stress E. For external load indicators, DEC showed significant correlations with every outcome (*p* < 0.05): total distance; m.min^−1^; sprint number; and distance in SZ1, SZ2, SZ3, SZ4, SZ5, and ACC. For internal load, DEC was also correlated with all indicators (*p* < 0.01) with mean HR%, and RPE (Figure 2). Finally, DEC showed significant correlations with sRPE (*p* < 0.001).

## 4. Discussion

The aim of this study was to investigate whether quantifying ACC and DEC could help to monitor the accumulated weekly training load in elite women soccer players. The results of the present study confirmed our hypothesis that these actions were sensitive enough to discriminate training load and were significantly associated with most of the training indicators usually monitored. Moreover, our results showed that a week with more ACC and DEC induced an increased fatigue as witnessed by the greater RPE and Hooper index.

The present investigation was conducted while considering two different weeks, in accordance with the staff, that were considered low and heavy weeks. Our results confirmed this a priori choice since most outcomes (except stress from Hooper and distance in SZ5) were significantly greater during the heavier week. Although not directly measured (for example, using a second CMJ at the end of each week), our results showed significant fatigue increases for most Hooper items (except stress). This finding therefore suggested that the two weeks under investigation were markedly distinct with additional work and fatigue during the heavy week when compared to the low week. In accordance with previous work, significant correlations were established between the two parameters under interest (i.e., ACC and DEC), TD [27], and RPE [28], as with most outcomes measured.

The correlation with TD can be explained by the fact that soccer-specific actions directly influence the number of ACC and DEC. These results are consistent with the significant correlation observed between ACC, DEC, and mean HR%. Indeed, it is known that the distance covered in different speed zones mainly influences HR [29,30]. In soccer, during training sessions, greater relative pitch area per player (obtained by dividing the total pitch area by the number of players) results in greater HR%, which can be attributed to fast running activities [31,32]. Furthermore, increasing the number of players and therefore, reducing the relative pitch area per player, would result in a greater number of ACC at low intensity, but not at moderate and high intensity. In order to elicit moderate and high intensity ACC, greater relative pitch area per player is required [33].

The conclusion that the number of ACC and DEC were influenced by pitch area was reinforced by examining training sessions. Indeed, during technical and physical training sessions, the coaching staff used only small-sided games to improve physical qualities, as well as technical skills. The small-sided games used were always 4 vs. 4 players, plus goalkeepers, on a 30 × 40 m field. This represents 150 m^2^ relative pitch area per player. As previously shown, this type of small-sided games elicits greater physiological demands compared to medium-sided games (5 vs. 5 to 8 vs. 8) and large-sided games (9 vs. 9 and more) [34,35,36] and a greater number of ACC and DEC that is comparable to match demand [37]. This can be explained by the fact that the relative pitch area per player used in this study is high compared to some 4 vs. 4 small-sided games. In fact, increasing the pitch area has been shown to expand the amount of movement “off-the-ball”, raising the physiological demands [38]. In addition, even though the pitch area per player is relatively high, it may not be enough to elicit high-speed running since no differences were obtained for Distance SZ5 and Sprints between the two weeks. Indeed, when reduced pitch size is used, limiting the use of high-speed running, ACC and DEC usually increase [39].

Therefore, the difference in readiness level at the end of both weeks could be explained by the number of ACC and DEC. As stated previously, the heavy week showed significant differences between HI Hooper S and HI Hooper E, while the low week load did not. This result is highlighted by most Hooper items. Sleep E, Fatigue E, and DOMS E were all greater at the end of the heavy week as opposed to the beginning. This would suggest that players’ readiness level was lower at the end of the heavy week. In accordance with previous authors, an increase of the accumulated weekly training load led to an increase of the HI [40]. This result could partially be explained by the content of the training sessions. In fact, during the low week, athletes completed two technical and tactical training sessions and one technical and physical training session. In contrast, during the heavy week, individuals completed one technical and tactical training session and two technical and physical training sessions. Indeed, small-sided games were implemented to realize the main work of the training sessions during the two monitored weeks. Small-sided games sessions are known to be more physically demanding than tactical sessions and lead to an increase in ACC and DEC numbers.

Taken as a whole, these results could also explain the correlation observed between ACC, DEC, and RPE. As previously shown, the elevated amount of ACC leads to an increased perception of exercise intensity [39].We could speculate that this correlation is attributed to the elevation of metabolites. Authors have previously demonstrated that an increase in ACC and DEC produced increased blood lactate and RPE [10]. ACC and DEC also correlated with HI Hooper E, Fatigue E, and DOMS E. In juxtaposition with previous findings, such observation could demonstrate that an increase in the number of ACC and DEC produced more muscle damage as attested by plasma creatine kinase [11]. This result could be explained by the predominance of eccentric muscle actions generated by DEC. In addition to a lower energy cost [9], DEC would produce greater muscle fatigue associated with muscle soreness. Additionally, ACC elicit a greater metabolic demand, as well as greater neural activation of working muscles, compared to constant-speed running [41]. Overall, these results may highlight the dominant role of ACC and DEC in the readiness level.

Some limitations could be acknowledged. Firstly, the present investigation was conducted while considering two different weeks inside the in-season training program. These weeks could obviously be influenced by the preceding weeks (training or competition). For that reason, we decided to consider training weeks within the first part of the championship. Secondly, the baseline players’ readiness statuses were controlled. Our results revealed similar CMJ, HI Hooper S, and Hooper items at the beginning of the two weeks. These similar baseline values suggested that players had comparable readiness status. The weeks could therefore be compared. Thirdly, the number of training sessions during the two weeks was quite low compared to other elite soccer teams. This reduced number was compensated by more intensive technical, tactical, and physical training sessions. Additionally, it is important to keep in mind that choosing different weeks or using different criteria to identify heavy and low weeks could have led to different results. Overall, the present study should therefore be duplicated in additional elite women soccer teams with greater training sessions per week.

## 5. Conclusions

Our study highlighted the need to quantify ACC and DEC alongside the indicators usually monitored in soccer. Indeed, these actions, even at sub-maximal speeds, are physiologically demanding. The indicators usually monitored, such as distance in different speed zones, total distance, and mean HR%, do not take into account ACC and DEC and may therefore underestimate the training load. Furthermore, this study is of particular interest as it highlights that considering these parameters enables practitioners to have a more global overview of athletes’ accumulated weekly training load. This could lead to a better understanding of the reasons why athletes become fatigued and give insight into neuromuscular, rather than only energetic, fatigue. Furthermore, practitioners should be aware that, when prescribing a training session, they should anticipate the number of ACC and DEC induced by their training session (for example, with the different forms of small-sided games). Indeed, this will directly influence the readiness level of the athletes and could affect their health status by increasing the subsequent injury risk.

## Figures and Tables

**Table 1:** Weekly training organization.

	AM	PM
Monday	Rest	Rest
Tuesday	Gym-based strength session	Field session
Wednesday	Rest	Field session
Thursday	Rest	Team meeting (video)
Friday	Rest	Field session
Saturday	Rest	Match
Sunday	Rest	Rest

**Table 2:** Values of monitored indicators during the low week and heavy week.

Indicator	Low Week	Heavy Week	P	d	95% CI
CMJ (cm)	27.31 ± 4.55	27.02 ± 4.11	0.329	0.29	−0.29 to 0.86
Mean HR (%)	62.53 ± 6.43	66.53 ± 6.69	0.010 *	−0.90	−1.56 to −0.20
TD (m)	3870 ± 870	5090 ± 620	<0.001 ***	−1.61	−2.46 to −0.72
m.min^−1^	56.72 ± 13.02	65.21 ± 6.69	0.019 *	−0.79	−1.43 to −0.12
Sprints (*n*)	6.79 ± 3.10	6.54 ± 2.81	0.732	0.10	−0.46 to 0.66
Distance SZ1 (m)	1483 ± 312	1917 ± 265	<0.001 ***	−1.39	−2.18 to −0.57
Distance SZ2 (m)	967 ± 285	1379 ± 274	<0.001 ***	−1.66	−2.54 to −0.76
Distance SZ3 (m)	655 ± 225	905 ± 231	<0.001 ***	−1.33	−2.11 to −0.53
Distance SZ4 (m)	301 ± 123	362 ± 100	0.031 *	−0.71	0.06 to 1.34
Distance SZ5 (m)	123.8 ± 60.5	154.7 ± 92.0	0.176	−0.41	−1.00 to 0.18
DEC (*n*)	31.52 ± 13.41	60.91 ± 14.42	<0.001 ***	−2.65	−3.87 to −1.41
ACC (*n*)	28.21 ± 11.93	56.14 ± 10.14	<0.001 ***	−2.66	−3.88 to −1.41
sRPE	210.0 ± 46.5	357.1 ± 49.51	<0.001 ***	−3.41	−4.92 to −1.88
RPE	3.31 ± 0.76	5.06 ± 0.68	<0.001 ***	−2.55	−3.74 to −1.34

Values are means ± SD. *p* values, effect sizes (d), 95% CI and significant differences are shown (* = *p* < 0.05, *** = *p* < 0.001). CMJ: Counter movement jump; HR: Heart rate; TD: Total distance; SZ: Speed zone; DEC: Deceleration; ACC: Acceleration; RPE: Rate of perceived exertion; sRPE: Session rate of perceived exertion.

**Table 3:** Values of Hooper index and Hooper items during the low week and heavy week.

	HI	Sleep	Stress	Fatigue	DOMS
**Low week**					
Hooper S	7.92 ± 3.32	1.92 ± 0.67	2.25 ± 0.87	1.83 ± 1.03	1.92 ± 1.24
Hooper E	8.83 ± 3.10	2.08 ± 1.16	2.33 ± 0.89	2.17 ± 1.34	2.58 ± 1.16
P (Cohen’s d/95% CI)	0.160 (−0.43/−1.01 to 0.16)	0.586 (−0.16/−0.72 to 0.41)	0.795 (−0.07/−0.64 to 0 49)	0.166 (−0.42/−1.01 to 0.17)	0.071 (−0.57/−1.18 to 0.04)
**Heavy week**					
Hooper S	8.17 ± 2.55	2.00 ± 0.85	2.33 ± 1.15	2.08 ± 0.90	2.00 ± 0.74
Hooper E	13.83 ± 4.22	3.08 ± 1.16	2.67 ± 1.23	3.25 ± 1.14	4.75 ± 1.42
P (Cohen’s d/95% CI)	<0.001 *** (−1.69/−2.58 to −0.78)	<0.001 *** (−1.37/−2.15 to −0.55)	0.463 (−0.22/−0.78 to 0.35)	<0.001 *** (−1.65/−2.53 to −0.75)	<0.001 *** (−1.71/−2.60 to −0.79)
**Low week/Heavy week: Hooper S**					
P (Cohen’s d/95% CI)	0.674 (−0.12/−0.69 to 0.44)	0.674 (−0.12/−0.69 to 0.44)	0.777 (−0.08/−0.64 to 0.48)	0.429 (−0.23/−0.80 to 0.34)	0.845 (−0.05/−0.62 to 0.51)
**Low week/Heavy week: Hooper E**					
P (Cohen’s d/95% CI)	<0.001 *** (−1.74/−2.65 to −0.81)	0.020 * (−0.78/−1.37 to −0.09)	0.226 (−0.33/−0.82 to 0.32)	0.002 ** (−1.20/−2.22 to −0.59)	<0.001 *** (−1.41/−2.33 to −0.65)

Values are means ± SD. *p* values, effect sizes (d), 95% CI and significant differences are shown (* = *p* < 0.05, ** = *p* < 0.01, *** = *p* < 0.001). Hooper S: Hooper at the start of the week; Hooper E: Hooper at the end of the week; HI: Hooper index; DOMS: Delayed onset muscle soreness.

**Table 4:** Correlations between ACC, DEC, and other indicators.

	ACC		DEC	
Indicator	Pearson’s r	P (95% CI)	Pearson’s r	P (95% CI)
HI Hooper E	0.427	0.037 * (0.029 to 0.708)	0.420	0.041 * (0.020 to 0.704)
Sleep E	0.270	0.202 (−0.150 to 0.607)	0.270	0.203 (−0.150 to 0.607)
Stress E	−0.358	0.086 (−0.666 to 0.053)	−0.327	0.118 (−0.646 to 0.087)
Fatigue E	0.422	0.040 * (0.022 to 0.705)	0.424	0.039 * (0.025 to 0.707)
DOMS E	0.548	0.006 ** (0.185 to 0.779)	0.531	0.008 ** (0.162 to 0.769)
HR%	0.495	0.014 * (0.114 to 0.749)	0.527	0.008 ** (0.157 to 0.767)
TD	0.771	<0.001 *** (0.533 to 0.896)	0.796	<0.001 *** (0.578 to 0.908)
m.min^−1^	0.612	0.001 ** (0.277 to 0.814)	0.654	<0.001 *** (0.341 to 0.837)
Sprint	0.479	0.018 * (0.093 to 0.739)	0.446	0.029 * (0.052 to 0.720)
Distance SZ1	0.657	<0.001 *** (0.345 to 0.838)	0.643	<0.001 *** (0.323 to 0.831)
Distance SZ2	0.582	0.003 ** (0.233 to 0.798)	0.629	<0.001 *** (0.302 to 0.823)
Distance SZ3	0.687	<0.001 *** (0.392 to 0.854)	0.744	<0.001 *** (0.487 to 0.882)
Distance SZ4	0.665	<0.001 *** (0.357 to 0.852)	0.687	<0.001 *** (0.392 to 0.854)
Distance SZ5	0.610	0.002 ** (0.274 to 0.813)	0.531	0.008 ** (0.162 to 0.769)
DEC	0.949	<0.001 *** (0.884 to 0.978)	-	-
ACC	-	-	0.949	<0.001 *** (0.884 to 0.978)
sRPE	0.758	<0.001 *** (0.511 to 0.889)	0.710	<0.001 *** (0.430 to 0.866)
RPE	0.730	<0.001 *** (0.463 to 0.876)	0.683	<0.001 *** (0.386 to 0.852)

Pearson correlation coefficients r, *p* values, 95% CI and significant differences are shown (* = *p* < 0.05, ** = *p* < 0.01, *** = *p* < 0.001). TD: Total distance; HR: Heart rate; Hooper S: Hooper at the start of the week; Hooper E: Hooper at the end of the week; HI: Hooper Index; DOMS: Delayed onset muscle soreness.

## Data Availability

The data presented in this study are available on request from the corresponding author.
